# Elevated miRNA-499 Levels in Early Phase of Non-ST Elevation Acute Coronary Syndromes Predict Increased Long-Term Risk of Major Adverse Cardiac Events

**DOI:** 10.3390/jcm13247803

**Published:** 2024-12-20

**Authors:** Dawid Miśkowiec, Ewa Szymczyk, Paulina Wejner-Mik, Błażej Michalski, Piotr Lipiec, Michał Simiera, Karolina Kupczyńska, Jarosław D. Kasprzak

**Affiliations:** Department of Cardiology, Medical University of Lodz, Kniaziewicza Street 1/5, 91-347 Lodz, Poland

**Keywords:** acute coronary syndromes, biomarkers, microRNA, mir-499, predictive value

## Abstract

**Background/Objectives**: Available data suggest the diagnostic potential of testing microRNAs (miRs) in myocardial infarction, but their prognostic value remains unclear. To evaluate the prognostic value of circulating miRs (miR-1, miR-21, miR-133a, miR-208 and miR-499) for predicting major adverse cardiac events (MACEs), including death, non-fatal myocardial infarction (MI) or cardiovascular rehospitalization, in patients with non-ST segment elevation acute coronary syndromes (NSTE-ACS). **Methods:** Our prospective, single-center, observational study included patients (pts) with NSTE-ACS admitted <24 h after symptoms onset and pts with confirmed stable coronary artery disease (SCAD) as controls. Relative expression of miRs was calculated, and subjects were categorized according to miRs expression on hospital admission into two groups (≤median and >median). **Results:** Overall, 103 NSTE-ACS (52 NSTEMI/51 UA) and 47 SCAD pts (median age 66 years, 67% male) were included. During the median 895 (581–1134) days of the follow-up, MACE occurred in 75 (50%) patients: 20 (13%) died, 28 (19%) presented with MI, and 65 (43%) were readmitted due to cardiovascular reasons. Incidence of MI, rehospitalization and MACE was significantly higher in pts with elevated (>median) miR-499 [MI: 34.3% vs. 7.3%; HR = 6.0 (2.8–12.7) for rehospitalization; 53.7% vs. 36.2%, HR = 2.3 (1.4–3.8) for MACE; 62.7% vs. 42%, HR = 2.4 (1.5–3.8)] for hospital readmission. In the Cox proportional hazards regression model, miR-499 expression above the median level [HR = 1.8 (1.1–3.1)], high-sensitivity cardiac troponin T [HR = 1.2 (1.02–1.5)], diabetes [HR = 1.7 (1.1–2.8)] and percutaneous intervention during hospital stay [HR = 2.1 (1.1–3.8)] were identified as independent predictors of MACE in long-term observation, even after adjustment for covariates. **Conclusions:** Elevated miR-499 level on hospital admission in NSTE-ACS is related to an increased rate of MACE in the 2.5-year follow-up.

## 1. Introduction

Acute myocardial infarction (AMI) remains a leading cause of morbidity and mortality in Western countries [1]. More than half of all acute cardiac events are classified as non-ST-elevation acute coronary syndromes (NSTE-ACSs). Patients presenting with NSTE-ACS pose a diagnostic challenge for physicians, because their presentation may vary from asymptomatic to severe hemodynamic or electrical instability or even cardiac arrest due to ongoing ischemia [1]. The most important aspects of managing patients with NSTE-ACS is the appropriate risk stratification to decide whether they will benefit from an either early invasive or pharmacological strategy. Even though several risk stratification tools were proposed, the search for novel biomarkers with high sensitivity and specificity for predicting adverse events to improve outcomes is warranted in this clinically challenging group of patients.

MicroRNAs (miRNAs) are emerging as pivotal modulators of cardiovascular development and disease. To date, more than 2000 human miRNAs have been cloned and sequenced. They are evolutionarily conserved, noncoding, short (~22 nucleotides) RNA molecules involved in post-transcriptional gene regulation by regulating messenger-RNA stability and function [2]. The studied miRNAs were found to regulate vascular smooth muscle cell differentiation (miR-133), apoptosis (miR-1, miR-133, miR-21, miR-499) endothelial function and angiogenesis (miR-1, miR-133), cardiomyocyte differentiation (miR-1, miR-133, miR-145, miR-208, miR-499) and repression of cardiac hypertrophy (miR-133) [3]. Some of them were reported to be related to the vulnerable plaques in the coronary arteries [4]. MiR-499 has been shown to have an important effect on the differentiation of cardiac stem cells into cardiomyocytes by targeting transforming growth factor β receptor 1 (TGFβR1) [5]. MiR-1 and -133a have been shown to play a key role in the development and differentiation of skeletal, cardiac and smooth muscles [6]. MiR-1 and miR-21 were associated with cardiac injury and cardioprotection, acting as anti-apoptotic and survival-promoting factors in different cells [7].

Recently, it has been suggested that selected miRNAs may serve as novel diagnostic markers of AMI. Their release may result not only from f-cell death and plasma membrane damage but also from an active cellular response to ischemia [8,9,10,11,12]. Therefore, they are promising valuable clinical biomarkers in the early diagnosis of myocardial infarction [3,8,9,11,12,13,14,15,16,17,18,19,20,21,22,23,24,25,26,27]. However, clinical studies on miRNAs expression in acute coronary syndromes are still limited and their findings are conflicting.

The aim of our study was to evaluate the differential expression and prognostic value of five circulating miRNAs—miR-1, miR-21, miR-133a, miR-208a and miRNA-499—in patients presenting with non-ST segment elevation acute coronary syndrome and control patients with stable coronary artery disease.

## 2. Materials and Methods

### 2.1. Study Design and Population

Our study was designed as a prospective, single-center, observational study. Unselected patients (adults of both genders) presenting to the emergency department (ED) with symptoms suggesting acute coronary syndrome (chest pain or its equivalent) with an onset or peak within the previous 24 h were recruited. The control group was composed of age- and gender-matched patients with stable coronary artery disease (SCAD) (Canadian Cardiovascular Society grading of angina pectoris II-III), confirmed by coronary angiography and defined as at least one coronary artery narrowing less than 75% (left main stem less than 50%), history of percutaneous coronary intervention or coronary artery bypass grafting (CABG). The SCAD patients were recruited from those electively admitted to the hospital for planned coronary angiography.

The patients with terminal kidney failure requiring dialysis or with an estimated glomerular filtration rate < 30 mL × min^−1^ × 1.73 m^−2^ were excluded from our study.

The study was carried out according to the principles of the Declaration of Helsinki, and the study protocol was approved by the Ethics Committee of the Medical University of Lodz in Poland (permission No.: RNN/9/13/KE; approval date: 15 January 2013).

### 2.2. Routine Clinical Assessment

The standard initial clinical assessment included medical history, physical examination, 12-lead resting electrocardiography (ECG), continuous ECG monitoring at ED and standard blood tests.

Blood was sampled twice in NST-ACS subjects at presentation and 3–4 h later. In the control group, blood was sampled once on hospital admission.

### 2.3. Final Diagnosis of Non-ST Myocardial Infarction

The final diagnosis of non-ST segment elevation myocardial infarction (NSTEMI) or unstable angina (UA) was performed with the use of a high-sensitivity cardiac troponin T (hs-cTnT) assay (Roche Diagnostics, Basel, Switzerland) and 0 h/3 h algorithm, according to current guidelines [1]. NSTEMI was diagnosed if at least one hs-cTnT was above the 99th percentile of the upper reference limit and after exclusion of other causes of symptoms. When serial hs-cTnT was below the 99th percentile of the upper reference limit, the unstable angina (UA) diagnosis was made.

### 2.4. Plasma miRNAs

Blood samples were collected twice in the NSTE-ACS group (on hospital admission and 3–4 h after) and once in the SCAD group.

#### 2.4.1. Isolation of miRNAs

Isolation of extracellular miRNAs from liquid samples was performed using Tri-Reagent LS (Sigma-Aldrich, St. Louis, MI, USA) together with the miRNeasy kit (Qiagen, Hilden, Germany). Briefly, 1 mL of Tri-Reagent LS was added to 0.2 mL of blood plasma, mixed by vigorous shaking for 10 s and then incubated for 10 min at RT to ensure complete dissociation of nucleoprotein complexes. Then, 5 pg of synthetic miRNA-39 from *Caenorhabditis elegans* (cel-miRNA-39) was added as a spike-in control for purification efficiency. After supplying 200 μL of chloroform, the mixture was vigorously shaken for 15 s and allowed to stand for 5 min at RT. Following centrifugation at 14,000× *g* for 20 min at 40 °C, total RNA was precipitated from the upper (aqueous) phase by adding 1.5 volumes of 100% ethanol. Purification of extracted total RNA was performed with miRNeasy columns (Qiagen) according to the manufacturer’s instructions. From the Qiagen columns, RNA was eluted in 40 μL of RNase-free water.

#### 2.4.2. miRNA Quantification

Reverse transcription was carried out on 5 μL of miRNA eluat in 15 μL reactions using the TaqMan^®^ MicroRNA Reverse Transcription Kit (Applied Biosystems, Foster City, CA, USA) according to the manufacturer’s instruction. miRNA quantification was performed using standard TaqMan^®^ MicroRNA Assays (Applied Biosystems): hsa-miR-1 (Assay ID: 002222), hsa-miR-21 (Assay ID: 000397), hsa-miR-208a (Assay ID: 000511), hsa-miR-133a (Assay ID: 002246), mmu-miR-499 (Assay ID: 001352) and cel-miR-39 (Assay ID: 000200) as a control. The 20 μL qPCR included 1.33 μL RT product, 10 μL TaqMan Universal PCR Master Mix and 1 μL TaqMan miRNA Assay (20×). The reactions were incubated in a 96-well plate at 95 °C for 10 min, followed by 40 cycles of 95 °C for 15 s and 60 °C for 1 min. All reactions were run in duplicate.

#### 2.4.3. Real-Time PCR Analysis

TaqMan PCR assays were performed on the 7900HT Fast Real-Time PCR System (Applied Biosystems) and analyzed using Sequence Detection System 2.3 software. Fold induction values (RQs) were calculated according to the equation 2^−ΔΔCt^, where ΔCt represents the differences in cycle threshold numbers between the target gene and spike-in control (cel-miR-39), and ΔΔCt represents the relative change in these differences between the examined and control groups.

### 2.5. Follow-Up and Clinical Endpoints

Patients were contacted 12 months after hospital discharge and before finalizing the study by telephone or by e-mail. The patients’ endpoints or events were checked by reviewing medical records or through follow-up telephone interviews. All studied patients received long-term follow-up.

The primary composite endpoint was defined as all-cause death or non-fatal myocardial infarction and/or hospitalization due to cardiovascular reasons.

### 2.6. Statistical Analysis

For categorical variables, data were presented as percentages, mean values with the standard deviation (SD) or the median with the interquartile range (IQR) for continuous variables, depending on their distribution. The normality of the data distribution was tested using the Shapiro–Wilk test. Student’s *t*-test for independent variables, the Mann–Whitney U-test or the Kruskal and Wallis test were applied to test the intergroup differences, depending on the variable distribution and group number. The categorical variable analysis was performed using the Chi-square test and Fisher’s exact test. Correlations analyses were performed with the use of the Spearman rank correlation coefficient.

The patients were categorized into two groups according to the median relative expression of particular miRNAs (<median and ≥median value) in order to perform survival analysis. The Kaplan–Meier survival curve with log-rank test and multivariable proportional hazard Cox regression (step forward selection method based on maximum likelihood ratio) analyses were applied. All *p*-values were 2-sided, and a *p*-value less than 0.05 was considered statistically significant. The overall statistical analysis was performed using MedCalc version 12.0 and STATISTICA version 13.0.

## 3. Results

### 3.1. Characteristics of Patients

Baseline characteristics of the studied groups are shown in Table 1.

**Figure 1:**
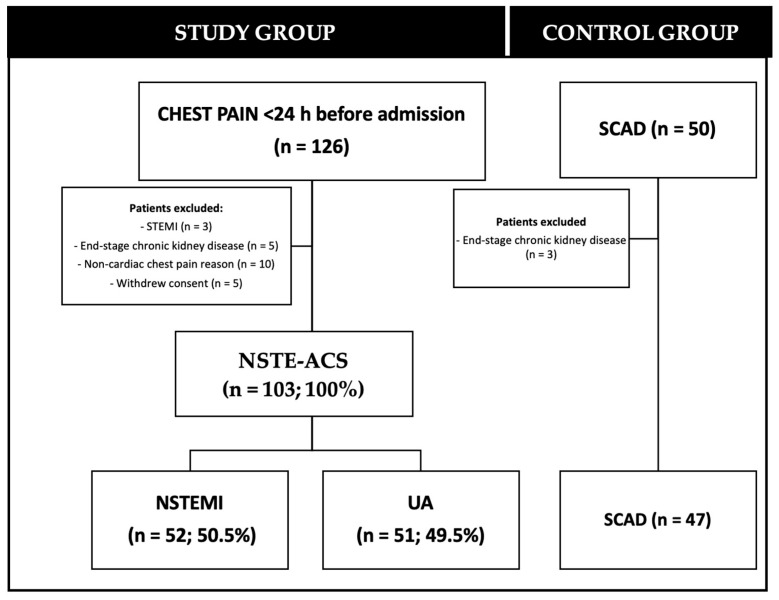
The study flow diagram. NSTE-ACS—ang. non-ST segment elevation acute coronary syndrome; STEMI—ST segment elevation myocardial infarction; NSTEMI—ang. non-ST segment elevation myocardial infarction; UA—ang. unstable angina; SCAD—ang. stable coronary artery disease.

One hundred and twenty-six patients with a clinical presentation suggesting acute coronary syndrome (chest pain or its equivalent), with the onset of index symptoms within 24 h, were screened. In total, 23 patients were initially excluded: ST-segment elevation MI (n = 3), 4th or 5th stage of chronic kidney disease (n = 5), non-cardiac chest pain reason (n = 10). The rate of withdrawal of consent for continued participation in the trial was 3.3% (5 patients in the NSTE-ACS group). NSTE-ACS as the final diagnosis was confirmed in 103 (NSTEMI in 52 patients, UA in 51 patients). The control group included 47 patients with stable coronary artery disease. Figure 1 shows the flow diagram of participation.

The groups were similar in age and gender (no significant differences between groups). The NSTEMI group demonstrated a higher rate of previous percutaneous coronary interventions (PCIs) than others (*p* < 0.05), whereas in the stable coronary artery disease group (SCAD), pulmonary disease (chronic obstructive pulmonary disease or asthma) was more frequent (Table 1).

### 3.2. Levels of miRNAs in NSTEMI, UA and SCAD

Among the five analyzed miRNAs, miR-1 (*p* < 0.001), miR-133a (*p* < 0.001), miR-208a (*p* = 0.03) and miR-499 (*p* < 0.001) levels were significantly higher in patients with NSTEMI than in those with SCAD. MiR-1 (*p* < 0.001), miR-133a (*p* < 0.001) and miR-499 (*p* < 0.001) levels were also significantly elevated in NSTEMI patients when compared to patients with finally diagnosed UA, whereas only miR-133a (*p* < 0.001) and miR-208a (*p* = 0.03) levels were significantly elevated in those with UA compared to SCAD (Table 2).

However, it should be noted that undetectable concentrations (Ct ≥ 40) were observed in the following percentage of patients: miR-1—three (2%) patients: two with SCAD and one with UA; miR-21—four (3%) patients: one with SCAD, two with UA and one with NSTEMI; miR-133a—one (1%) patient with SCAD; miR-208a—82 (55%) patients: 32 with SCAD, 33 with UA and 17 with NSTEMI. Bearing in mind the issue of undetectable miRNAs values in further analyses, we have handled these missing values (Ct ≥ 40) by imputation of the median value for miR-1, miR-21 and miR-133a. The imputed values were the median for the particular group (SCAD, UA, NSTEMI) and only for prognostic analysis of long-term adverse events. For the individual comparison (initial expressions of analyzed miRNAs), the missing values were not imputed. Because of a high percentage (65%) of patients with miR-208a levels under the detection limit, we decided to exclude these cases from the overall analysis.

### 3.3. Prediction of Major Adverse Cardiac Events (MACEs) Using miRNAs

During a median follow-up of 895 (581–1134) days, the composite primary endpoint (MACE) was observed in 75 (50%) patients. There were 20 (13.3%) deaths, 28 patients (18.6%) presented with new non-fatal MI, and 65 patients (43.3%) were readmitted due to cardiovascular reasons.

To investigate the utility of the analyzed miRNAs for predicting adverse cardiovascular events, Kaplan–Meier curves analysis was performed on the data for all studied patients (SCAD, NSTEMI and UA). We demonstrated the cutoff values of miRNAs using the median value of relative expression (for patients with NSTEMI and UA, it was the median of the mean relative expressions from two blood samples—on admission and 3–4 h after admission) and separated the patients into two groups (above or equal to the median value and below the median value).

**Figure 2:**
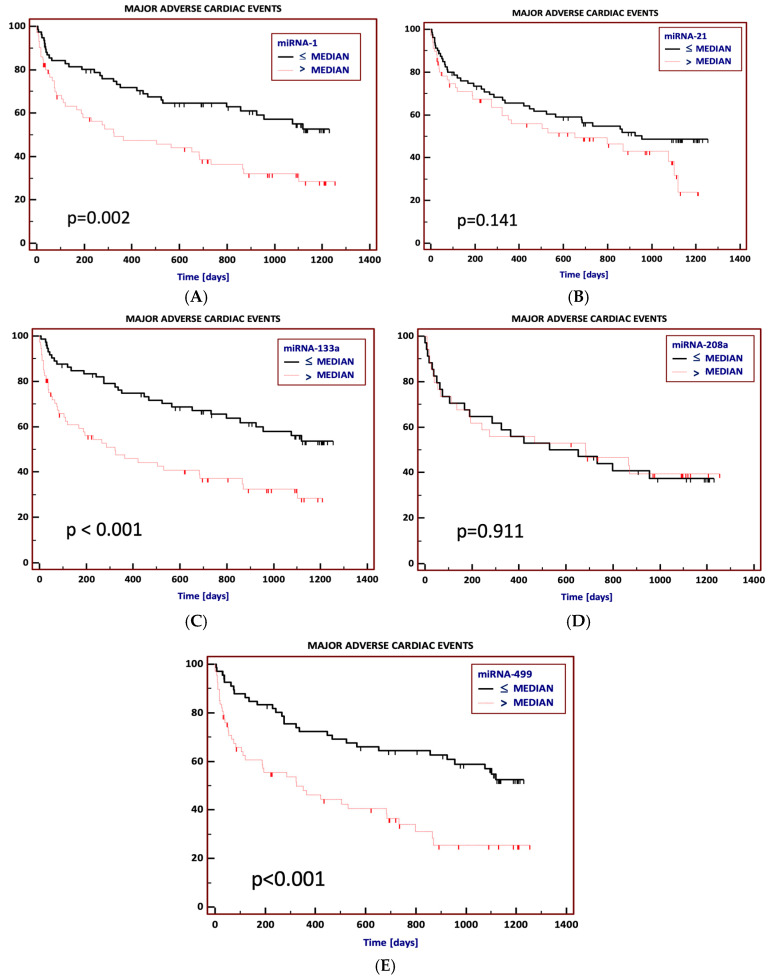
(**A**–**E**) Kaplan–Meier event-free survival curves for freedom from major adverse cardiac events in long-term observation according to particular miRNA levels on hospital admission (above or equal to the median value and below the median value: (**A**) miRNA-1; (**B**) miRNA-21; (**C**) miRNA-208a, (**D**) miRNA-133a; (**E**) miRNA 499.

During the follow-up, the incidence of MACE was significantly higher in patients with elevated levels of miR-1 [59.3% vs. 42.1%, HR = 2.0 (1.3–3.3), *p* = 0.002], miR-133 [59.5% vs. 40.5%, HR = 2.3 (1.4–3.7), *p* < 0.001] and miR-499 [62.7% vs. 42%, HR = 2.4 (1.5–3.8), *p* < 0.001] on hospital admission (Figure 2A–E).

Non-fatal myocardial infarction during the follow-up occurred more frequently in patients with elevated miR-1 [29.6% vs. 9.6%, HR = 3.8 (1.8–8.0), *p* < 0.001], miR-133a [33.8% vs. 4.1%; HR = 11.3 (5.3–23.9), *p* < 0.001] and miR-499 [34.3% vs. 7.3%; HR = 6.0 (2.8–12.7), *p* < 0.001] (Figure S1A–E).

Moreover, the risk of readmission to hospital due to cardiovascular reasons was significantly higher in patients with initially elevated miR-1 [52.1% vs. 35.5%, HR = 2.1 (1.3–3.5), *p* = 0.002], miR-133a [54.1% vs. 32.4%, HR = 2.6 (1.6–4.3), *p* < 0.001] and miR-499 [53.7% vs. 36.2%, HR = 2.3 (1.4–3.8), *p* < 0.001] (Figure S2A–E).

There were 20 deaths (13.3%) during the long-term (median 895 days) follow-up. Of all the studied miRNAs, only patients with elevated miR-208a had a significantly lower risk of death [29.3% vs. 5.9%, HR = 0.21 (0.1–0.7), *p* = 0.03] (Figure S3A–E).

In the Cox proportional hazards regression model, miR-499 expression > median level (HR = 1.8; 95% CI 1.1–3.1), high-sensitivity cardiac troponin T (HR = 1.2; 95% CI 1.02–1.5), diabetes mellitus (HR = 1.7; 95% CI 1.1–2.8) and percutaneous intervention (PCI) during hospital stay (HR = 2.1; 95% CI 1.1–3.8) were independent predictors of MACE in long-term observation, even after adjustment for covariates (other miRNAs levels, hypertension, hyperlipidemia, body mass index, gender, PCI, multivessel coronary disease, prior MI, CK-MB mass levels on admission—Table 3).

The prognostic accuracy of miR-1 [AUC = 0.63; 95% CI 0.54–0.71], miR-133a [AUC = 0.64; 95% CI 0.55–0.72] and miR-499 [AUC = 0.66; 95% CI 0.58–0.74] for MACE was comparable to the accuracy of hs-cTnT (AUC = 0.69; 95% CI 0.61–0.77), *p* > 0.05, for all comparisons between hs-cTnT and all miRNAs.

None of the analyzed miRNAs was identified as an independent predictor of long-term mortality.

## 4. Discussion

In our prospective study, we have demonstrated not only differential expression of miR-1, miR-133a and miR-499 in patients with NSTE-ACS and SCAD, but also their robust predictive value for major adverse cardiac events. To our knowledge, the present study is one of the first to show that particular miRNAs can provide significant prognostic information in ACS and SCAD patients. Indeed, miR-1, miR-133a and miR-499 were associated with MACE during long-term follow-up, and miR-499 even after adjustment for age, gender, hs-cTnT and other cardiovascular risk factors. None of the other five investigated miRNAs was associated with the outcome. Our results support the presented circulating miRNAs as novel cardiovascular biomarkers, which could improve risk stratification in cardiac patients.

Published studies on miRNAs, including the molecules studied in our research, i.e., miR-1 [10,28,29,30,31], miR-133a/b [10,18,21,29,30,32,33] and miR-499 [10,11,18,23,24,25,26,27,28,29,31,32,34,35,36,37], suggest that they could serve as diagnostic markers across different stages of cardiovascular disease progression [3]. In general, their expression increases with the advancement of disease—in healthy individuals, they are often undetectable, whereas in patients presenting with acute myocardial infarction, extremely high levels are observed [17,37,38]. Although none of the miRNAs chosen for our study presented a robust predictive value for long-term mortality, three of them—miR-1, miR-133a and miR-499—were related to the composite endpoint (MACE). We hypothesized that this finding could reflect the cardiovascular disease progression, supporting the findings of Chen et al. They have shown a positive correlation between miR-499 levels and the Gensini score [39], and therefore, determine long-term outcomes. Our study is one of a few that relate miRNAs in NSTEMI patients to clinical findings in the prospective follow-up. Further research is required to determine the additive prognostic value of their deregulation to better predict new clinical adverse events.

Evidence is emerging on the diagnostic value of different miRNAs for distinguishing between ACS and SCAD patients, but only limited data are available regarding the relationship of miRNAs and cardiovascular outcomes. Several exploratory small studies provided mixed results, and recently, larger studies on the prognostic value of circulating miRNAs have been published [40]. Karakas et al. [41], the authors of a recent and probably the largest study, conducted in a cohort of 430 patients with ACS and 682 with SCAD, concluded that seven other miRNAs (miR-19a, miR-19b, miR-132, miR-140-3p, miR-150, miR-186 and miR-210) have strong predictive value for cardiovascular death, with area under the receiver operating curves (AUCs) up to 0.76 [41]. Widera et al. found that elevated miR-133a and miR-208b were significantly associated with mortality in patients with ACS [30], whereas in our study, miR-133a correlated only with the composite endpoint (MACE), and contrarily, elevated miR-208a was related to lower long-term mortality. In their study, Widera et al. reported a 2.5-fold higher risk of death in the fourth-quartile miR-133a expression, when compared with patients in the first three quartiles [30]. The levels of miR-133a predicted mortality even after adjustment for age and gender, but not after adjustment for admission concentrations of hs-cTnT [30]. Also, in the study by Ke-Gang et al., miR-133a levels had a significant association with the risk of endpoint events at 24 months, even after adjustment for covariates [21]. However, another study by Devaux et al. found no significant difference in miR-133a and miR-499 expression in individuals who died versus in survivors, whereas only miR-208b levels were significantly higher [32]. In their work, the prognostic accuracy of miR-208b for 30-day mortality was borderline significant, with AUC 0.67 (95% CI 0.52–0.81), while all other studied miRNAs (including miR-133a and miR-499) were not significantly predictive for early death. Notably, only 19.4% of all studied patients in the work of Devaux et al. were finally diagnosed with AMI [32], and that diversity of the analyzed cohort may significantly affect the results of our study, where all patients have been diagnosed with coronary artery disease. Similarly, in our study, none of the analyzed miRNAs predicted long-term mortality, except for miR-208a, but considering the high percentage of undetectable levels (65%) of miR-208a and the applied method of missing data handling, this finding should be interpreted with caution.

We also replicated the findings of previous studies that presented an increased miR-499 level in AMI patients [11,13,16,19,29,30,31,35,37,42,43]. However, this is one of the first reports to show that, in addition to their diagnostic value, elevated miR-499 levels are significantly associated with MACE in long-term follow-up, even after adjustment for hs-cTnT and other cardiovascular risk factors. These findings are in contrast to results of a study conducted by Goretti et al. on a population of 510 consecutive patients with AMI followed up for 6 years [34]. They observed that neither miR-208b nor miR-499 was a significant predictor of long-term mortality; however, the overall long-term (6 years) mortality rate in this study was only 12.5% [34]. In our study of a 2.5-year follow-up duration, 13.3% of the patients died, indicating a higher disease burden in our NSTE-ACS patients population. In another report on 155 elderly patients (mean age > 80 years) with NSTEMI, Olivieri et al. found that patients with miR-499-5p levels on admission exceeding the median value had a two-fold higher risk of death within 12 months (HR = 2.05; 95% CI 1.15–3.81), which is consistent with our findings and suggests an improved prognostic performance of miR-499 when added to cardiac troponins in NSTE-ACS patients management. Also, Wang et al. in their study demonstrated that combination of miR-499, miR-22 and GRACE scores had a stronger discrimination power for MACE occurrence, with a sensitivity of 100% and specificity of 79%, supporting our findings [11].

Even though our study is relatively small and single-center, its findings suggest that miR-1, miR-133 and especially miR-499, combined with established risk factors and validated biomarkers, may help identify patients with a worse long-term prognosis and recognize those who could benefit from an early invasive and aggressive approach.

### Study Limitations

Our study has some limitations that need to be addressed. Firstly, there was no kinetic analysis of the circulating miRNAs levels signature. We were surprised to find that in our study group, fatal cardiovascular events were uncommon, which can reflect effective early treatment strategies. Another limitation lies in the fact that miRNAs are often circulating in a very low concentration, and even highly sensitive analytic methods appear to be ineffective at detecting low levels of miRNAs. Moreover, sometimes certain miRNAs are even totally absent from the circulation [44]. This could often result in missing values for the measurement of miRNAs, or the values can also be missing due to a technical error. However, in order to reduce potential bias, we made miRNAs measurements in duplicate. It allowed us to improve, at least partly, the precision of miRNAs quantification, and we could distinguish missing data because of low expression from technical errors. Nonetheless, the major limitation of our study is the fact that in 65% of patients, particularly those with SCAD, levels of miR-208a were undetectable. As it was mentioned in Section 3, we handled missing miR-208a values by excluding these data, which could have led to a loss of power and meaningful differences; these finding could be misleading and should be interpreted with caution. Another source of potential bias could be the normalization approach with the use of cel-miR-39. Since it is not incorporated in lipid complexes, microvesicles or proteins, variations in extraction of miRNAs from them are not taken into consideration [44]. Therefore, an ideal approach would involve the use of endogenous miRNAs, guaranteeing better data robustness and taking analytic failures during the RT-qPCR into account [44,45].

## 5. Conclusions

MiR-1, miR-133a and miR-499 levels are increased in NSTE-ACS patients and provide prognostic information independent of established risk indices. In particular, miR-499 levels were shown to be an independent predictor for major cardiovascular events in long-term follow-up, even after adjustment for hs-cTnT and other comorbidities.

## Figures and Tables

**Table 1:** Characteristics of studied groups.

Variable	Stable Coronary Artery Disease (n = 47)	NSTE-ACS (n = 103)		*p*-Value
		NSTEMI (n = 52)	UA (n = 51)	
Male, n (%)	16 (34%)	14 (27%)	19 (37%)	0.52
Age, years	65.3 (10.6)	66.8 (11.1)	67.7 (12.2)	0.47
BMI, kg/m^2^	28.6 (24.8–30.5)	28.2 (26.0–30.3)	28.4 (25.0–30.5)	0.92
Cardiovascular risk factors				
Diabetes mellitus, n (%)	19 (40%)	20 (38%)	14 (27%)	0.34
Hypercholesterolemia, n (%)	40 (85%)	38 (73%)	44 (86%)	0.17
Hypertension, n (%)	40 (85%)	44 (85%)	46 (90%)	0.66
Smoking, n (%)	15 (32%)	27 (52%)	17 (33%)	0.07
Positive CAD family history ^a^, n (%)	2 (4%)	8 (15%)	8 (16%)	0.14
Stroke, n (%)	5 (11%)	5 (10%)	10 (20%)	0.26
TIMI Risk Score, median (IQR)	-	4.5 (3–6)	4 (3–5)	0.06
GRACE Risk Score	-	159.0 (33.8)	139.4 (29.1)	0.002
AF, n (%)	5 (11%)	6 (12%)	7 (14%)	0.89
Prior MI, n (%)	13 (28%)	11 (21%)	18 (35%)	0.28
Prior PCI, n (%)	21 (45%)	9 (17%)	23 (45%)	0.003
Prior CABG, n (%)	3 (6%)	3 (8%)	4 (8%)	0.91
Lung disease ^b^, n (%)	6 (13%)	2 (4%)	0 (0%)	0.02
Laboratory values on admission				
eGFR, mL × min^−1^ × 1.73 m^−2^	81.5 (26.2)	83.5 (24.3)	74.7 (29.1)	0.18
NT-proBNP, ng/L	403 (98–890)	1192 (257–2813)	336 (110–661)	0.01
CRP, mg/dL	2.5 (1.0–5.0)	4.0 (1.7–26.4)	2.4 (0.9–8.2)	0.06
TC, mg/dL	155 (130–184)	194 (154–214)	160 (147–199)	0.03
LDL, mg/dL	81 (67–115)	116 (81–141)	84 (71–108)	0.01
HDL, mg/dL	53 (44–68)	42 (34–53)	49 (40–62)	0.01
TG, mg/dL	91 (67–148)	109 (92–160)	136 (53–166)	0.02

^a^ Defined as myocardial infarction in direct blood relatives (male < 55 years; female < 65 years); ^b^ Chronic obstructive pulmonary disease and/or asthma. NSTE-ACS—non ST-elevation acute coronary syndrome; NSTEMI—non ST-elevation myocardial infarction; UA—unstable angina; CAD—coronary artery disease; AF—atrial fibrillation; Prior MI—prior myocardial infarction; Prior PCI—prior percutaneous coronary intervention; Prior CABG—prior coronary artery bypass grafting; eGFR—estimated glomerular filtration rate calculated using the Cockcroft–Gault formula; NT-proBNP—N-terminal pro-brain natriuretic peptide; CRP—C-reactive protein; TC—total cholesterol level; LDL—low-density lipoprotein level; HDL—high-density lipoprotein level; TG—triglycerides level; Kruskal–Wallis test or Chi^2^ test was used to compare presented data.

**Table 2:** Mean relative expression of miRs and routine myocardial infarction biomarkers in studied groups.

Biomarker	Stable Coronary Artery Disease (n = 34)	NSTEMI (n = 20)	UA (n = 16)	p_1_	p_2_	p_3_
miR-1	4.68 (2.17–9.37)	20.04 (7.56–68.88)	4.49 (2.43–8.02)	<0.001	<0.001	1.0
miR-21	1.88 (1.12–2.94)	2.41 (1.21–4.55)	2.60 (1.37–4.28)	0.32	1.0	0.19
mir-133a	4.54 (2.25–7.22)	39.94 (20.02–405.94)	10.91 (6.09–19.38)	<0.001	<0.001	<0.001
miR-208a	5.03 (3.33–15.49)	19.15 (6.41–42.05)	28.45 (5.94–51.27)	0.03	1.0	0.03
miR-499	1.76 (1.19–2.68)	12.91 (3.41–63.33)	3.23 (1.60–4.21)	<0.001	<0.001	0.11
hs-cTnT, ng/mL	0.013 (0.010–0.021)	0.405 (0.160–1.60)	0.015 (0.009–0.033)	<0.001	<0.001	1.0
CK-MB mass, ng/mL	2.40 (1.80–3.40)	13.00 (7.88–37.92)	2.22 (1.82–3.10)	<0.001	<0.001	1.0
h-FABP, ng/mL	-	52.2 (20.69–108.7)	21.9 (9.7–57.6)	-	0.04	-

hs-cTnT—high-sensitivity cardiac troponine T; CK-MB mass—creatine kinase isoenzyme; h-FABP—heart-type fatty acid binding protein; p_1_—for differences between stable coronary artery and NSTEMI patients; p_2_—for differences between NSTEMI and UA patients; p_3_—for differences between stable coronary artery and UA patients; NS—not significant.

**Table 3:** Results of Cox proportional hazards regression model for predicting major adverse cardiac events in long-term observation.

Variable	HR	95% CI	*p*
miRNA-499 > median	1.80	1.05–3.08	0.02
hs-cTnT	1.24	1.02–1.50	0.04
Diabetes mellitus	1.72	1.06–2.78	0.03
PCI at hospital stay	2.07	1.13–3.82	0.02

Abbreviations—see Table 1 and Table 2. The covariates used in the Cox proportional hazards regression model: miRNA-1 > median, miRNA-133a > median; miRNA-21 > median, miRNA-208a > median, miRNA-499 > median, age, gender, body mass index, left ventricular ejection fraction, CK-MBmass, hs-cTnT, diabetes mellitus, hypertension, hyperlipidemia, smoking, prior myocardial infarction, prior PCI, atrial fibrillation, heart failure, PCI at hospital stay.

## Data Availability

The data that support the findings of this study are available from the corresponding author, Dawid Miśkowiec (dawid.miskowiec@gmail.com), upon reasonable request.

## Supplementary Materials

The following supporting information can be downloaded at: https://www.mdpi.com/article/10.3390/jcm13247803/s1, Figure S1: Kaplan–Meier event-free survival curves for freedom from rehospitalization due to cardiovascular reasons in long-term observation according to particular miRNA levels on hospital admission; Figure S2: Kaplan–Meier event-free survival curves for freedom from non-fatal myocardial infarction in long-term observation according to particular miRNA levels on hospital admission; Figure S3: Kaplan–Meier survival curves for overall survival in long-term observation according to particular miRNA levels on hospital admission.
