# Supplementary material for: Elevated miRNA-499 Levels in Early Phase of Non-ST Elevation Acute Coronary Syndromes Predict Increased Long-Term Risk of Major Adverse Cardiac Events
Source: J Clin Med. 2024 Dec 20;13(24):7803. doi: 10.3390/jcm13247803 (PMC11727993; doi:10.3390/jcm13247803)
Supplement: Supplementary file 1 [file jcm-13-07803-s001.zip › mRNAs PREDICTIVE VALUE - FIGURE S2.pdf]

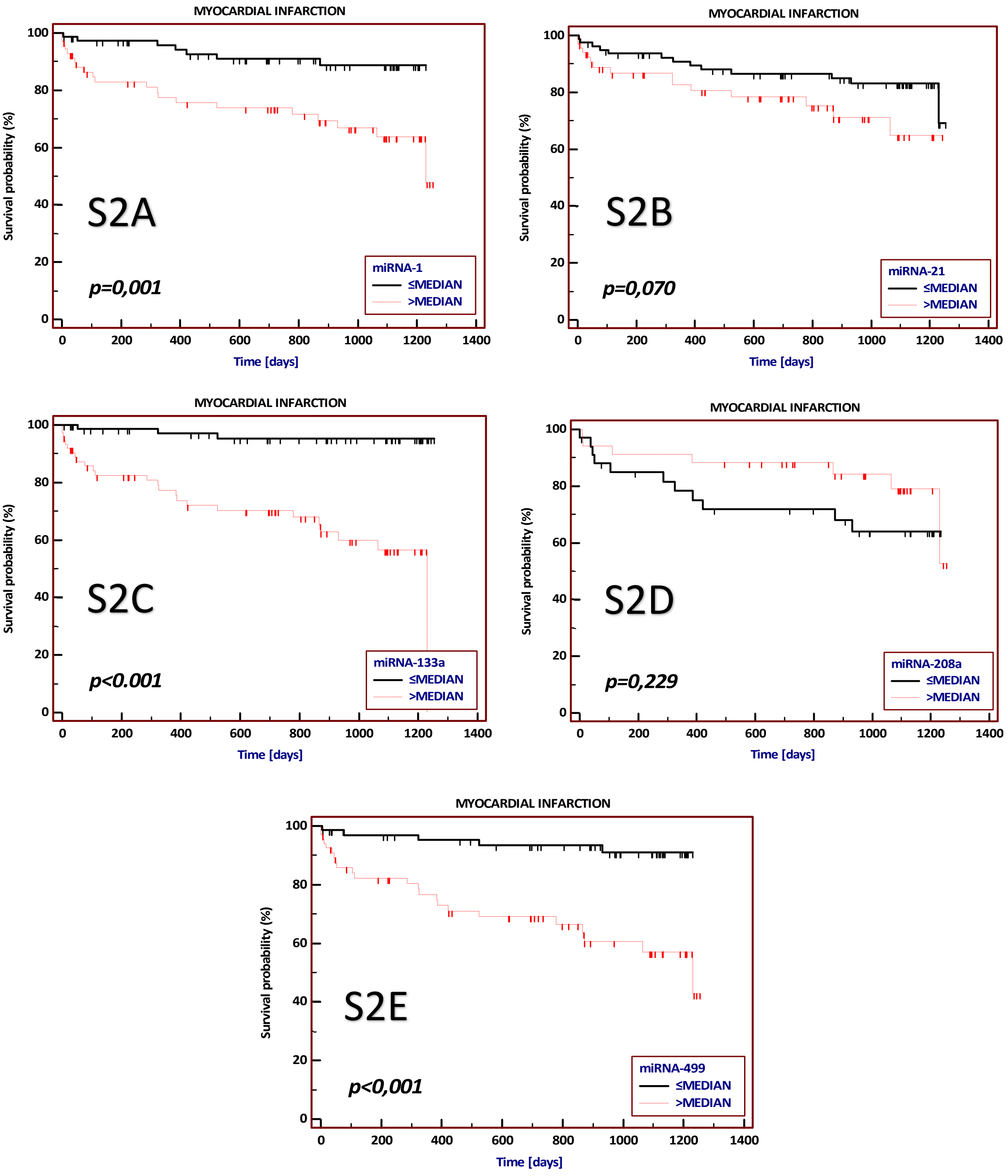

Figure S2A-E. *Kaplan-Meier event-free survival curves for freedom from non-fatal myocardial infarction in long-term observation according to particular miRNA levels on hospital admission (above or equal to the median value and below the median value;* ***S2A*** *– miRNA-1;* ***S2B*** *– miRNA-21;* ***S2C*** *– miRNA-208a,* ***S2D -*** *miRNA-133a;* ***S2E*** *– miRNA 499).*
